# Mechanisms of embodiment

**DOI:** 10.3389/fpsyg.2015.01525

**Published:** 2015-10-15

**Authors:** Katinka Dijkstra, Lysanne Post

**Affiliations:** Department of Psychology, Faculty of Social Sciences, Erasmus University Rotterdam, Rotterdam, Netherlands

**Keywords:** embodied cognition, sensorimotor simulation, memory, cognition, online embodiment, offline embodiment

## Abstract

This paper is a critical review of recent studies demonstrating the mechanism of sensorimotor simulation in different cognitive domains. Empirical studies that specify conditions under which embodiment occurs in different domains will be discussed and evaluated. Examples of relevant domains are language comprehension (Tucker and Ellis, 1998), autobiographical memory (Dijkstra et al., 2007), gestures (Alibali et al., 2014), facial mimicry (Stel and Vonk, 2010), and problem solving (Wiemers et al., 2014). The focus of the review is on supporting claims regarding sensorimotor simulation as well as on factors that modulate dynamic relationships between sensorimotor components in action and cognitive domains, such as expertise (Boschker et al., 2002). This discussion takes place within the context of currently debated issues, specifically the need to specify the underlying mechanisms of embodied representations (Zwaan, 2014; Körner et al., 2015).

## Introduction

More than two decades after the grounding problem in symbol theories was brought up (Harnad, 1990) embodied cognition approaches have gained a stronghold in the study of cognition (Dijkstra and Zwaan, 2014). Since the first notion that cognition is grounded in perception and action (Glenberg, 1997), an abundance of empirical studies have demonstrated support for this groundedness (see for example Glenberg et al., 2013; Dijkstra and Zwaan, 2014).

Recently, the need to take stock of what all these studies contribute to the concept of grounded and embodied cognition has been expressed (Willems and Francken, 2012). Moreover, discontent with the current state of affairs has been noted. For example, opposing results in different studies have been interpreted as supporting embodied cognition approaches, indicating that the predictions may be too general to be falsified (Willems and Francken, 2012). Also, boundary issues have been raised regarding what phenomena embodied cognition approaches may or may not be able to explain (Mahon and Caramazza, 2008). For example, the issue whether, or to what extent, abstract symbols are also grounded in action and perception is a recurring topic in the debate on embodied versus disembodied approaches. Abstract concepts generally do not have physical or spatial referents, which renders a direct mapping of an abstract concept with a sensorimotor domain problematic at least (Mahon and Caramazza, 2008; Dijkstra et al., 2014).

This criticism does not stand on its own but is complemented by constructive proposals to counter the potential “erosion of the embodiment concept” (Willems and Francken, 2012) and to get out of the “impasse” regarding the discussion around the grounding of language comprehension (Zwaan, 2014). These proposals converge on the issue that the focus of current research should not be on supporting either embodied or disembodied accounts but on *how* sensorimotor systems and cognitive processes interact. The *pluralist view of cognition* proposed by Zwaan (2014) entails that abstract and grounded symbols contribute differently to language comprehension depending on how language is embedded in the environment in which it is used. In this view, research should focus on what the representations consist of and assess when and how they interact (Zwaan, 2014). Others proposals claim that research on embodiment lacks explanative power (Körner et al., 2015), should specify the conditions under which conditions certain phenomena do or do not occur (Willems and Francken, 2012), and integrate existing knowledge regarding embodiment to describe its underlying mechanisms (Körner et al., 2015).

This review is written within the scope of these proposals to more specifically assess conditions under which embodiment occurs or not and to gain insight into underlying mechanisms of embodiment. To accomplish this, studies are drawn from various domains in research in which embodiment effects have been found under clearly defined conditions and that demonstrate the mechanism that underlies these embodiment effects. The selection of studies discussed in this review is by no means exhaustive but illustrates how a limited number of claims that are empirically supported can contribute to a deeper understanding of embodiment effects. “Embodiment” is defined here broadly as the effect that the body or parts of the body (movement, position) can have on cognition or vice versa.

## Mechanisms of Embodiment—Sensorimotor Simulation

Recent reviews on embodied cognition have focused on establishing mechanisms of embodiment (Körner et al., 2015), describing relevant domains in embodiment effects (Dijkstra and Zwaan, 2014), such as language comprehension (Fischer and Zwaan, 2008), or stipulating a theory on embodied simulation (Gallese, 2007, 2009), or grounded cognition (Barsalou, 2008, 2010). A common element in their reviews is that sensorimotor simulation is considered to be a core element in cognitive processing.

Most recently, the idea of sensorimotor simulation as one of the main mechanisms underlying embodiment, has been developed further into specific ways in which this sensorimotor simulation takes place (Körner et al., 2015). The first claim is that the perception of a stimulus automatically triggers the simulation of reenactment with it. Actions are facilitated when they match the simulated actions and impeded when there is a mismatch between the two. Secondly, simulation may be blocked by a concurrent task that involves the same sensorimotor resources. The third claim is that simulation may also work offline, whereas the fourth claim entails that simulation depends on previous experiences and skills. Two other claims are discussed later on in this review.

In this review, both the mechanism of sensorimotor simulation as the claims derived from it are discussed in the context of research domains that illustrate the conditions under which embodiment occurred or failed to occur. Some of these domains have already been studied extensively, such as language comprehension, whereas fewer studies have been dedicated to other domains, such as autobiographical memory, gestures, expertise, facial mimicry, and problem solving. The review will remedy this omission by devoting more attention to these domains.

## Sensorimotor Simulation in Language Comprehension

The idea of sensorimotor simulation is that there are neural correlates between the content of what is being read and represented (i.e., action words) and the areas in the brain being activated (i.e., actions). Embodied cognition research on language comprehension has focused on examining the simulations being formed when reading sentences that are compatible with certain actions that are performed (Glenberg and Kaschak, 2002; Fischer and Zwaan, 2008; Kaschak et al., 2014). In one study, participants read sentences describing actions toward the body (John gave you a pencil) or away from the body (I closed the drawer) and had to make sensibility judgments regarding the content of the sentence by moving their hand toward or away from their body by pressing a button. Participants responded faster when the direction implied in the sentence (i.e., toward the body) was congruent with their own action (Glenberg and Kaschak, 2002). This supports the claim of congruence effects in sensorimotor simulation facilitating performance for congruent over incongruent conditions.

Other studies have shown how simulation is prevented from happening and how this affects cognitive processing. If the neural system is already engaged with a task at the time that a similar task requires a response and also involves the same neural circuits, interference will occur and embodiment is impeded. Consequently, there will be no facilitation of a congruent bodily manipulation on cognitive processes. This has been demonstrated in a study in which participants listened to sentences that implied upward or downward motion and concurrently watched a display on a screen that would scroll upward or downward (e.g., the cat climbed the tree; Kaschak et at., 2005). Participants had longer response times to decide whether the sentence made sense or not when sentence content and visual display were congruent than when they were incongruent. It was not possible to simulate the motion they represented from the sentence as they were processing the same motion in a different task. In the second experiment (Kaschak et at., 2005), participants responded faster to sentences when the visual stimulus concurrently showed motion in the opposite direction as was implied by the sentence content. Apparently, when neural circuits are already engaged for the motion part of the experiment, it is not possible to simulate sentence content that requires a mental representation of motion information at the same time. Thus, interference occurred for congruent but not for incongruent mappings.

The studies discussed above illustrate how and when the simulation mechanism operates and when it does not. They also support the claim that the perception of stimuli triggers their simulation when they match and the claim that simulation may be blocked by a concurrent task that involves the same sensorimotor resources. Compatibility effects are only present when neural circuits are available for simulation of congruent stimuli materials. If not, interference for processing congruent stimuli materials occurs. The third claim that simulation can work offline is also supported by empirical research and is described in more detail next.

Sensorimotor simulation may happen offline (Niedenthal et al., 2005; Körner et al., 2015) in the absence of a bodily state or action. Research has shown that retrieving an object from memory, and thinking or imagining an object generates responses in the body that show similarities to the responses that would occur if the object were present (Brouillet et al., 2010). Such an offline embodiment effect was demonstrated in a study conducted by Tucker and Ellis (1998). In this study, lists of words were presented to participants. Half the words represented small (penny) and half the words represented large objects (beach ball) and these words could be either natural or artificial. Participants indicated whether the object was natural or artificial with a joy stick and either had to apply a full grasp of the joystick or a precision grasp between thumb and forefinger. The results indicated that congruence in the grasp response with the type of object resulted in faster responses (small object-precision grip) than when this congruence was absent (large object-precision grip). Apparently, participants have a similar response in the absence of the object as when the object would have been actually present.

Another study demonstrating offline embodiment under congruent conditions of sensorimotor simulation was a language comprehension study (Pecher et al., 2009) in which participants responded whether a picture of an object occurred in the sentence preceding the picture. Previous studies have demonstrated that participants tend to simulate the shape of the object, being slower in the yes response of the object having occurred in the sentence when the shape does not entirely match the implied shape of the object in the sentence than when the object matches closely in shape (Stanfield and Zwaan, 2001; Zwaan et al., 2002). These were online effects. In the study by Pecher et al. (2009), however, the matching or mismatching pictures were presented at the end of the experiment when all sentences had been presented or after a 45-min delay. In both retention conditions, a match effect was found. Simulation of relevant aspects of the stimuli (shape) from memory should have occurred because only this mechanism can explain the faster responses under congruent (match) than under incongruent (mismatch) conditions.

The studies discussed above demonstrate sensorimotor simulation while processing the stimuli or considerable time after these stimuli have been processed or reconstructed from memory. Sensorimotor simulation affects the speed with which responses are made and requires that relevant aspects of the stimuli (for example shape or motion direction) are congruent in order to transpire unless neural circuits are already activated in a concurrent sensorimotor simulation and therefore blocking the simulation from happening. These results illustrate what favorable conditions are for sensorimotor simulation. Simulation happens under favorable conditions, such as compatibility, without the requirement that a bodily state or action is present at the same time. Simulation is hindered, however, when the same sensorimotor resources engaged in one task, are also needed for a second, concurrent task, suggesting that simulation may be a default mechanism that is constrained by how resources are engaged. More compatibility effects have been demonstrated in research on language comprehension (see for an overview Fischer and Zwaan, 2008; Kaschak et al., 2014) but it exceeds the purpose of this review to mention them all. Importantly, we aim to illustrate how certain claims regarding the sensorimotor simulation also operate in different domains, such as gestures.

## Sensorimotor Simulation in Gestures

According to the Gesture as Simulated Action (GSA) framework (Hostetter and Alibali, 2008), gestures emerge when premotor activation exceeds a certain threshold and spreads to motor activation. This premotor activation is evoked by embodied (sensorimotor) simulations, which occur, for instance, while thinking about a task that would be facilitated by the use of gestures, such as pointing. The motor activation that results from exceeding the threshold is a gesture. This way, gestures express embodied simulations.

Gestures do not only express embodied simulations, but even have a causal role in the sense that gestures highlight perceptual and motor information which are consequently more likely to be incorporated in reasoning (Alibali et al., 2014). Specifically, the information conveyed in gestures influences the listeners’ reasoning, because they refer to present and absent entities through pointing gestures and representational gestures, respectively, which help the listener to comprehend what the speaker is saying. Besides literally seeing what the speaker means because of the pointing gestures, the referential gestures can influence the readers simulations through activation of the motor system, but they can also cause the listener to mimic the speakers’ gestures which allows the gestures to influence the listeners’ reasoning the same way they influenced the speaker. This influence of gestures on simulations proposed in the GSA framework is therefore in line with the idea of sensorimotor simulation as a major mechanism underlying embodiment as proposed by Körner et al. (2015).

Indeed, several studies suggest that perception of gestures automatically triggers its simulation and that simulation may also work offline. A study by Cook and Tanenhaus (2009), for example, showed that speakers’ co-speech gestures while explaining a problem solving task affected listeners’ behavior when solving the problem on a computer (later in time, i.e., offline), that is, the mouse trajectories resembled the observed gestures. There is even evidence that speakers’ gestures activates listeners’ motor systems (i.e., automatic simulation; Ping et al., 2014). This study also showed that, in line with the claims made by Körner et al. (2015), actions are facilitated when they match the simulated actions and impeded when they mismatch. That is, the initially found positive effect on reaction times for pictures after congruent sentences from observing gestures disappeared when listeners moved their arms and hands. In contrast, the effect did not disappear when participants moved their legs and feet, indicating that the speakers’ gestures indeed activated the listeners’ motor system, that is, the part of the motor system that is involved in gesture related body parts, the arm and hands (Ping et al., 2014).

There seems to be, however, more to gesturing than sensorimotor simulation. Pouw et al. (2014) focus on the mechanism of offloading onto the environment, a claim on embodiment stipulated by Wilson (2002). According to Pouw et al.’s (2014) theory, gestures are external placeholders for internal processes. This offloading on the environment allows more internal processes to take place at the same time. Their point is illustrated by the following two examples. First, gesturing as if one actually rotates an object during a mental rotation task (e.g., Chu and Kita, 2008) can reveal information that is otherwise difficult to mentally compute and provide a physical platform to support internal processes (Pouw et al., 2014). Secondly, pointing gestures can aid internal cognitive processes by helping to keep track of counting (Delgado et al., 2011) or mental calculations (Hatano et al., 1977; Hatano and Osawa, 1983). Thus, Pouw et al. (2014) argue that gestures have a cognitive function, because they are used for and support cognition when the costs of mental computation are high (either by internal or external constraints).

Now, within the scope of the current paper, we ask what can the interaction between gestures and memory tell us about the underlying mechanisms of embodiment? Are gestures an externalization of cognition (Pouw et al., 2014) or are the relationships between gestures and cognition bidirectional, with one influencing the other and vice versa (Alibali et al., 2014)? Even though these two theories are not mutually exclusive, empirical research has mainly shown support for the latter claim (Beilock and Goldin-Meadow, 2010; Hostetter and Alibali, 2010; Post et al., 2013).

Positive effects of gestures on children’s memory were found in a substantial number of studies on mathematics (Broaders et al., 2007; Cook et al., 2008), memory of a fictional story (Stevanoni and Salmon, 2005), and word learning (Tellier, 2008; see Macedonia and Von Kriegstein, 2012, for a review). Word learning in a foreign language was also facilitated by gesture observation for adults in a study by Kelly et al. (2009). These results are in line with the current ideas about embodiment. In the different studies, gesture production and observation seem to reflect and even elicit sensorimotor simulations. Because it is theorized that these simulations automatically emerge, gestures should be an effective way to improve memory. Indeed, memory was improved when participants used gestures (Kelly et al., 2009).

The studies discussed above demonstrated positive effects of gestures on learning and memory. But there are also circumstances under which gestures do not enhance learning (Beilock and Goldin-Meadow, 2010; De Nooijer et al., 2013; Post et al., 2013). Does that mean there is no embodiment in those cases? Or do those studies perhaps even provide evidence that embodiment is not characterized by sensorimotor simulations? Not necessarily. Beilock and Goldin-Meadow (2010) had participants gesture while explaining the Tower of Hanoi task. Their gestures reflected disk size and weight, as they used one-handed gestures when referring to the light disks and two-handed gestures when referring to the heavy disks. In a second task, weight was switched in a way that was less compatible with the gestures that were made earlier (i.e., small disks were heavier than large disks). Performance for participants in the gesture condition declined, indicating that the gestures actually represented the weight of the disks. Thus, in this case a detrimental effect of gestures is actually revealing sensorimotor simulations (i.e., embodiment).

Other studies in which negative effects of gestures were found shed light on the boundary conditions of sensorimotor simulation. For example, Post et al. (2013) found that, for children with poor general language skills, gestures increase cognitive load and hamper learning when imitated online during the first encounter with new material. For these children, sensorimotor simulation, and thus embodiment, was prevented by the cognitive overload they experienced. De Nooijer et al. (2013) found that gestures do help when imitated during either learning or testing, but not when imitated during both. It is not entirely clear what caused the effects found by the Nooijer et al., but it is clear that gestures are not always beneficial for memory.

There is also empirical evidence of the influence of cognition on gestures. Gesture frequency during descriptions of images of dot patterns was influenced by participants’ physical experience with the patterns (Hostetter and Alibali, 2010). Participants gestured more when they had specific physical experience with the patterns than when they had only learned visually. The experiments show that this was not due to a decrease in verbal rehearsal or motor priming. Hostetter and Alibali conclude that gestures occur when people talk about thoughts that involve action simulations.

In sum, within the domain of gestures, the interaction of action and cognition is bidirectional. Gestures are a form of actions that can influence learning and memory and this is interpreted as support for embodied cognitive processes (Hostetter and Alibali, 2008; Pouw et al., 2014). In line with the claims made by Körner et al. (2015), the described gesture studies support the idea that perception can automatically trigger sensorimotor simulations, that actions can be facilitated or hampered depending on congruency with the simulated actions, and that simulations also work offline. However, there are boundaries as to how far this embodiment of gestures reaches, because gestures are not always supportive for learning, for example when cognitive resources are overloaded (Post et al., 2013).

The domain of gestures is after language comprehension the second cognitive domain that illustrates how sensorimotor simulation operates, under which conditions, and what the boundaries are. Another domain in which relevant aspects of the body can influence memory through simulation is autobiographical memory.

## Sensorimotor Simulation in Autobiographical Memory

A simulation view of autobiographical memory entails that modality-specific states of perception, action, and introspection that were activated when an event was experienced, are activated again when the experience is represented at a later point in time (Niedenthal et al., 2005, 2014; Dijkstra et al., 2007; Dijkstra and Zwaan, 2014). If autobiographical memory is a simulation and reconstruction of the original experience along with the relevant perceptual, sensorimotor and affective components of the experience, facilitation of the retrieval process should occur if these components of the original experience are triggered when the retrieval process is initiated (Dijkstra and Zwaan, 2014).

Support for this prediction was found in a study in which participants retrieved autobiographical memories in body-congruent and body-incongruent positions relative to that of the original experience (Dijkstra et al., 2007). Participants were not only faster retrieving the memory when prompted after assuming a body-congruent position (talking about a previous visit to the dentist while being reclined in a chair) than in a body-incongruent position (talking about this dentist visit while standing up with the legs out and the hands in the waist) but also retained the memory better after a period of 2 weeks when they were asked to talk about the memories they retrieved 2 weeks earlier. This study demonstrates sensorimotor simulation under congruent conditions of body position both during retrieval and long-term retention tasks. It seems as if body position is a sensorimotor component of the original experience that acts as an embodied cue to facilitate the reconstruction of the original experience. The ease of retrieval is reflected in faster retrieval times to access a relevant (i.e., body congruent) memory and even strengthens the memory trace so that body-congruent memories are remembered better at a later point in time.

This study supports two of the claims that were discussed earlier, compatibility effects when there is a correspondence between the stimuli condition (body position) and its simulation (the body position that was relevant during the original experience). The other claim is that the study demonstrates offline embodiment because experiences from the past are being reconstructed rather than experiences that happen at this moment. The study also supports the claim regarding embodiment by Wilson (2002) that offline cognition is body based, specifically for memories that are records of spatiotemporally localized events that are relived by the person who remembers them. Studies demonstrating congruence effects with a body manipulation thus provide strong support for claims regarding sensorimotor simulation.

Do these simulations extend to more indirect relationships between bodily states and autobiographical memory simulations? For example, the mapping of certain actions, such as moving hands “up” or “down” with certain emotions, such as “positive” for “up” and “negative” for “down” illustrate such an indirect relationship. This mapping is an example of a conceptual metaphor, abstract concepts that have metaphorical associations with actual experiences (Dijkstra et al., 2014). Conceptual metaphors arise from a pattern of associations of concrete experiences (cheering, jumping out of joy) with certain body movements (upward movement). Conceptual metaphors, such as “positive is up” can be understood in terms of concrete concepts and experiences, such as the times that you cheered when your favorite soccer team scored a goal, or the times you slumped in your seat when the other team scored against your team. Based on the conceptual metaphor that maps verticality with valence, the prediction can be made that the retrieval of positive or negative autobiographical memories should be facilitated when an up or down action is performed, but only under movement-valence congruent conditions (up and positive, down and negative).

This facilitation was demonstrated in a study involving body movement and emotional memory retrieval (Casasanto and Dijkstra, 2010). Participants retrieved autobiographical memories to prompts while moving both hands upward to deposit a marble in each hand in two containers that were placed in a higher location, or downward into containers below. The idea behind the study was that an upward movement triggers the “up is positive” metaphor and facilitates retrieval of positive memories, while a steady downward movement triggers the “down is negative” metaphor and facilitates retrieval of negative memories. The to-be-retrieved memories, were either positive or negative (Tell me of a time you felt proud of yourself/Tell me of a time you felt ashamed of yourself) in Experiment 1, and neutral (Tell me of an event that happened yesterday) in Experiment 2. In Experiment 1, reaction times to congruent (positive is up) and incongruent (positive is down) movement/valence memories were assessed. Participants were faster retrieving memories in congruent than incongruent trials. In the second experiment, participants again moved their hands but first retrieved memories to neutral prompts silently and then retold the memories afterward when their hands did not move. They were more likely to retell memories they later judged as positive when they moved their hands upward.

Just as in the previous study with body position (Dijkstra et al., 2007), body movement seemed to facilitate access to the memory itself by activating a relevant aspect of the experience, the emotion that was experienced at that time. This was an indirect trigger, however, because the emotion arose as a result of the mapping of a motor action and the associated emotion with that action. Both studies demonstrate the role of offline sensorimotor simulation under congruent conditions. The last study also supports the claim by Körner et al. (2015) that simulation may play a causal role in processing emotion. Only the activation of the mapping between valence (“positive is up”) and the vertical movement of the hands (up) explains why participants have better access to emotion-movement congruent memories and attribute a certain emotion to memories when they move their hands a certain way.

A similar embodiment effect demonstrating the mapping of body position with valence was demonstrated in a study by Riskind (1983). Participants were instructed to be either in an upright position and smile or in a slumped position with a downcast expression and their head and neck down. While being in this position, they had to retrieve unpleasant and pleasant experiences. The results indicated that participants were faster in retrieval of these experiences in body position-valence congruent condition (upright and pleasant or slumped and unpleasant) compared to body position-valence incongruent conditions. Again, research demonstrated that manipulations of the body affect cognitive processes under specific conditions: those of body-valence congruence.

A last study that contributes to our insight into sensorimotor simulation in autobiographical memory does not involve the retrieval of emotional experiences but the generation of emotion words, such as “disappointment” and “pride” that are associated with previous emotional experiences in which the word was relevant (Oosterwijk et al., 2009). Given the association of positive words with up movements and negative words with down movements, changes in body posture (straight up or slumped) were expected depending on the emotion that was elicited. Participants’ height was measured (with a hidden camera) during the word generation procedure which would give an indication of the posture demonstrating pride (upright posture) or disappointment (slumped posture). The results showed that participants indeed changed their body position to a lower, more slumped position when disappointment words compared to the body position during the generation of pride words (Oosterwijk et al., 2009).

Other than the previous study, this study illustrates a more subtle embodiment effect. Body position was influenced by the simulated sensation that was triggered by the valence of the presented words. A major difference with the autobiographical memory studies, discussed above, is that the effect of perceiving a valenced stimulus triggered the simulation of the valence-matching body position, not the other way around as in the previous studies. Such bidirectional effects were also demonstrated in the discussion of research on gestures. A similarity with the autobiographical memory studies on valence was that all three studies illustrate the claim that simulation plays a causal role in processing emotion. A stimulus or action that is congruent in valence with the simulated stimulus or action can be simulated more readily and easily than when these favorable conditions are absent. Autobiographical memory retrieval and sensorimotor processes tap into long-term memory stores of experiences that are being simulated when triggered appropriately. In other domains, the accumulation of motor experiences builds up to a certain level of motor fluency and a knowledge base that can be tapped into when being triggered. Individuals, who have done this over a long period of time, can tap into this store more easily and efficiently than those who have not. They are considered to be experts.

## Sensorimotor Simulation: Expertise

What happens when a person has performed complex motor movements and motor sequences so many times that a high level of expertise has been reached? From an embodied cognition perspective, if someone has extensive experience with in an action domain, such as tennis, more grounded representations will be activated when playing or talking about the sport compared with a novice. This fits with the claim by Körner et al. (2015) that automatic simulation depends on previous experiences and skills. Experts are likely to form a full-blown first person mental simulation of the described actions (with many grounded representations) whereas a domain-novice would form shallow, word-like representations instead (Sutton and Williamson, 2014). The motor expertise that has been developed would then facilitate any processing and memory of expertise-related information that is presented to this expert. Several studies have demonstrated support for this assumption.

In one study, expert climbers were tested to see if they would remember climbing routes of wall elements better compared to novices (Boschker et al., 2002). Expert climbers have built up motor fluency of specific climbing movements and should therefore remember these routes better. In addition, they should notice elements of the climbing environment as potential holds for grasping and establishing a route for ascend or descend to a greater extent than novices. The results supported the assumption that experts remembered the climbing routes with the affordances that the wall elements offered whereas the more inexperienced climbers tended to focus on structural features of the climbing wall and did not remember the routes as well. Expertise thus facilitates the way to take in and remember expertise-related information that results in superior performance on cognitive tasks. Here, the sensorimotor system involves an accumulation of experiences that feed into the cognitive domain.

There is another relevant aspect of expertise when considered from an embodied cognition perspective, which may actually hinder performance on certain tasks, and this is loss of attentional control. Expert skills are built up over a substantial period of time, resulting in competencies that are part of procedural knowledge and no longer require explicit attentional control (Beilock et al., 2004). Consequently, if actions have to be performed with unlimited time available, novice learners in a domain should benefit from this whereas it may actually harm an expert because control processes may come into play for a task that only requires procedural skills (Beilock et al., 2004).

These effects of expertise were demonstrated when experts imagined actions that were within their expert motor repertoire (Beilock and Gonso, 2008). Novice golfers had lower accuracy scores in putting when speed was stressed in the instruction and they had to actually make a putt, or imagine putting a certain sequences of actions. In contrast, expert golfers performed better on actual and imagined putting when time was more limited because they tapped into their proceduralized skill under conditions of time pressure but allowed conscious control when more time was available which impaired their performance. Imagery appeared to serve the function of action readiness in these experts. This is similar to the step-by-step unfolding of the action itself and involves the same cognitive and motor parameters. When speed is no issue, other elements become important in the imagery process of experts that involve explicit control of the skill, which gets in the way of the planning of actual steps.

Motor expertise may therefore enhance memory performance and performance accuracy because complex patterns are practiced frequently and readily available. However, superior memory performance in experts tends to be limited to expertise-related information relative to everyday information and novices when stimuli were encoded through observation, or enactment (Dijkstra et al., 2008). The knowledge and experience base are specific to the domain of expertise and do not necessarily transfer to other domains.

Because the action patterns that form the basis of expert performance are overlearned, motor expertise may also lead to reconstruction bias (Barsalou, 1999) in tasks that may cause some confusion as to whether an item tapping into this fluency was encountered before or not. As a result, it may be more difficult to differentiate between patterns that were or were not presented or imagined previously. This false recognition bias was examined by (Yang et al., 2009) who assessed the effect of the expert skill of typing on recognition rates of letter dyads that would normally be typed with the same finger (j and h with the index finger) or with different fingers (j and l), reflecting motor fluency among expert typists. The idea was that the activation of action plans that are associated with the different letter dyads, such as dyads reflecting higher motor fluency could lead to decision errors on a recognition task among experts but not novices. Experts have more consistent mappings between certain letters and how they type them. The results supported this assumption. Skilled typists recognized different-finger letter dyads that were not presented earlier incorrectly as having been presented before more frequently than novices. Recognition memory was influenced by the covert simulation of repeated action patterns among experts. As a typist, you cannot help but covertly simulate the motor action of typing letters when you are merely processing them visually. Therefore, you think you saw letter dyads before because you have a covert motor trace of activating these letters. In other words, your motor fluency gets in the way of simply performing the cognitive task.

These studies on the role of expertise show how sensorimotor simulation operates in the domain of expertise. The activation of previous experiences relevant for an expert task results in the availability of relevant knowledge for a particular task. This knowledge can be utilized in the performance of motor and memory tasks. If other information is allowed to enter the system when there is ample time available or if processing information in one modality allows for covert simulation of action patterns that are part of motor fluency, expertise can actually get in the way of performance in the cognitive domain. What we learn from these studies is that there are different aspects of expertise that play a differential role in the interaction between sensorimotor systems and cognitive domains. These have to be taken into account when examining the role of expertise from an embodied cognition perspective.

The studies discussed so far were chosen because their results support claims regarding sensorimotor simulations. Language comprehension is facilitated under congruent stimulus-simulation conditions and hindered when the neutral circuits needed for simulation are already engaged with another task. Gestures can be considered as a way to offload information into the environment reducing working memory load and increase availability of cognitive resources for internal cognitive processes. Bodily cues, such as body position, help the reconstruction process of the original experience when retrieving an autobiographical memory. Expertise intensifies the interactions between action and cognitive domains. Repeated action patterns allow experts to take in and remember more relevant aspects of a scene than novices (Boschker et al., 2002) because they have learned to perceive their environment in a different manner. All these effects operate through sensorimotor simulation under congruent conditions and both online and offline. However, once motor fluency has been developed in experts, it cannot be undone easily which suggests that performance may include activated yet incorrect information from a motor repertoire, forming a bias in memory processes.

In the preceding sections, research on offline embodiment effects was discussed in the domains of language comprehension, gestures, autobiographical memory, and expertise. Simulation may occur in the absence of an object, in a later stage of stimuli processing, when retrieving an event from the past, and as an accumulation of previous experiences. Another domain in which offline embodiment effects can be explained with sensorimotor simulation, is problem solving.

## Sensorimotor Simulation: Problem-solving

Embodied research on problem-solving often demonstrates the use of physical or imagined simulation to facilitate finding the solution for a task. A well-known example in this respect is a study by (Kirsh and Maglio, 1994) in which participants have to rotate and flip falling block shapes quickly to make them fit with the surface and the blocks they fall on. Participants use the strategy of actual rotation of the blocks in order to determine the best fit rather than mental computation to solve the problem. This fits with the idea of sensorimotor simulation and the facilitating role of the body in the simulation process (Dixon et al., 2014).

An example of such simulation is a study that employed a gear-system problem. Participants have to predict the turning direction of the final gear in a series based on the turning direction of the gear that drives the force to the system. Participants typically solve this problem by employing their body, that is, by manually simulating the movement with their hand of each gear that follows the previous one (Dixon et al., 2014). This manual simulation then helps participants to discover the higher order solution that applies to all these problems which is the insight that alternation occurs of the gear direction.

Simulation based on indirect but commonly occurring mappings between abstract concepts and concrete experiences, as we saw in the studies in the autobiographical memory domain has also been demonstrated in the domain of problem solving. Apart from the “positive is up,” mapping, other mappings exist, for example in relation to the mental number line, “many is up” or “few is left.” They can also be activated with sensorimotor manipulations and facilitate processing of information if the mapping is present. Numerical magnitude can be represented both horizontally, with left representing a smaller quantity than right (Pinhas and Fischer, 2008), and vertically, with up representing a larger quantity than down (Shaki and Fischer, 2012). We stack coins into piles with higher piles indicating higher quantities and numbers are usually written horizontally with lower numbers being presented on the left and higher numbers being presented on the right.

Wiemers et al. (2014) examined the activation of these two representations, horizontal and vertical, in a mental arithmetic task. Participants performed addition and subtraction tasks while making upward and downward movements, movements to the right and left, or no movement at all with their right arm in Experiment 1. They solved more problems under movement/magnitude congruent than incongruent conditions. In Experiment 2, they did the same arithmetic task but this time the arithmetic problem (and not their arm) moved in the directions described above. Again a compatibility effect of movement with the spatial representation of the task (up and addition with down and subtraction) was shown. This study demonstrates the idea of sensorimotor simulation through compatibility of actual and perceived movement in order to solve the task. Moreover, earlier experiences with arithmetic and the representation of magnitude were activated when the magnitude-spatial mapping occurred which resulted in facilitation of the response.

Werner and Raab (2014) also investigated the mapping between horizontal spatial representations and the abstract concept of magnitude but in a different type of problem solving task. The authors used the water-jar problem which requires participants to obtain a required volume of water when they are given certain empty jars for measure. Participants were primed to a left or right gaze direction in a perception task to mentally compare full jars either to the left or the right of a similar empty jar. This should bias them to either the left or right when being presented with the water jug problem and after they sorted marbles from outer bowls inward (plus group) or the other way around (minus group). Participants indeed demonstrated a spatial bias in gaze direction to the right for the plus group and to the left for the minus group without there being differences in overall problem solving ability.

A similar gaze design was used by (Thomas and Lleras, 2007) who manipulated gaze behavior in participants prior to them being exposed to a problem solving task, known as the Duncker radiation task. Participants have to destroy a tumor in a patient on a computer screen with lasers while keeping the tissue around the tumor healthy. The solution to this task is to have multiple laser beams fire at low intensity at the tumor from various locations around the tumor so that the convergence of these beams can destroy the tumor, yet keep the tissue intact. Participants whose gaze behavior was manipulated to make saccadic eye movements between the tumor and the surrounding locations, which hints at the solution, were more successful in solving the problem later than participants who were instructed to fixate their gaze on the tumor. Here, the practiced eye movements (moving in and out versus fixed) facilitated later problem solving by activating the sensorimotor simulation to tackle the problem.

A last study demonstrating sensorimotor simulation in a problem solving task involved spatial working memory (Thomas, 2013). Again, the underlying assumption was that movement with arms or eyes may embody the solution to an insight problem through simulation. A second assumption was, however, that actions will only affect problem solving when the representations of these actions are active in working memory. If these representations are (no longer) active, no effect will occur. Participants tried to solve the Duncker radiation problem, but different from the study discussed above, participants had to occasionally perform a visual tracking task. Moreover, they held a spatial (a grid filled with dots) or verbal stimulus (a string of digits) active in working memory at the same time. It was expected that holding a spatial stimulus in memory would engage spatial working memory resources and therefore interfere with the problem solving task, even if the eye movements are directed at various locations around the tumor. The results showed that being assigned to an embodied-solution condition (observing different colored stimuli from different locations around the tumor toward the tumor and out again) indeed was not sufficient for better problem solving performance. Only if they also held a verbal stimulus active in working memory did they solve the Duncker problem after fewer attempt intervals. They also did this faster than participants who were in the same embodied-solution condition but also held a spatial stimulus active. In other words, representations in spatial working memory affected problem solving even if participants were visually primed toward a solution of the problem. This demonstrates the simulation blocking effect that we encountered in the discussion of language comprehension research. If neural circuits are already engaged with a concurrent task, simulation is impeded or blocked.

The studies discussed from the domain of problem solving also support the claims regarding sensorimotor simulation. Under specific conditions of congruence as long as neural circuits are not engaged beyond capacity, sensorimotor simulation may aid problem solving. This simulation may be a physical or mental simulation and can take place during the task or afterward. It may also apply to the last domain to be discussed in this review, facial mimicry. Research conducted within this domain also supports the last claim from Körner et al. (2015) that has not been discussed as of yet, and that is the claim that simulation is important in social interactions.

## Sensorimotor Simulation: Facial Mimicry

Facial mimicry is the imitation of facial expressions by a person who is observing these expressions in another person. This commonly happens when emotions are expressed on the face (smile or frown), usually in a situation in which someone is communicating with someone else but also when observing pictures or videos of other persons who display an emotion on their face. The mimicry of these emotions on the face is the simulation of what one observes. How does this happen? When individuals observe actions and emotions of others, the same neural circuits are engaged as when the same actions and emotions would be experienced personally (Oosterwijk and Barrett, 2014). We therefore understand the feelings and emotions of another person through the engagement of neural circuits based from a recreation of these feelings in ourselves (Niedenthal et al., 2005). From an embodiment perspective, this is an interesting notion because it illustrates how embodiment is a shared rather than an individual phenomenon and it fits with a sensorimotor simulation account that presupposes a causal role regarding emotion processing and is important in social interactions (Körner et al., 2015).

Researchers on facial mimicry agree that emotions are grounded in online bodily states that arise from a context-specific experience in which the emotion is evoked and distributed across multiple regions of the brain (Niedenthal et al., 2014). Facial mimicry then is the way how the sharing of emotion becomes apparent for the other (Niedenthal et al., 2014). Facial mimicry can help to strengthen social bonds between people because a speaker receives through the facial expression of the listener the feedback that the other understands what is being communicated. If the expression is emotional, facial mimicry may signal the other is feeling the same way. This benefit of understanding the other may not be limited to understanding how the other person is feeling but extend to understanding what this person is communicating.

Empirical support for these assumptions comes from research manipulating how an emotion can be expressed on the face (Niedenthal et al., 2001; Oberman et al., 2007; Stel and Vonk, 2010). An emotional expression on the face can either be induced by placing a pen between the teeth, or blocked by placing a pen between the lips to block the expression of smiling. In one study employing the emotion-blocking procedure, some participants held a pen in the mouth between the lips to hinder the expression of smiling whereas other participants could display their emotions freely. When instructed to detect changes in facial emotion expression in morphed pictures (from sad to happy) participants were slower to recognize the emotion change when their ability to display the emotion was blocked than participants who did not have this restriction (Niedenthal et al., 2001). In another emotion-blocking study, participants placing pressure on a pen, showed an impaired ability to recognize happy faces relative to participants who could show facial expressions without being hindered in their expression and thus engaging the relevant muscles (Oberman et al., 2007). Both studies demonstrated better performance on a cognitive task when participants were able to freely express their emotions on the face relative to a condition in which the expression was blocked. Other than the studies discussed above in which simulation was impeded because of engagement of neural circuits with a concurrent task, here simulation is physically blocked by limiting the muscles to contract to express emotion.

These effects of manipulations of the face can also be the result of more invasive procedures, for example when getting the first Botox injection. People have these injections to eliminate wrinkles from their forehead for a certain period of time. A by-effect is that at the peak of the effectiveness of the injection, the corrugator muscle that normally is involved in frowning does not participate in the frowning after Botox is injected. This non-participation of the muscle was used in a study in which participants read sentences with angry, sad, and positive content right before and 3 weeks after the Botox injection when the effectiveness of the injection reaches a peak (Havas et al., 2010). The results indicated that reading times were slower after the injection than before but only for the sentences with negative content. Because participants were no longer able to frown due to the paralysis of the muscles in the corresponding area, facial feedback of expressing the emotion to emotion-relevant neural circuits was disrupted and processing of negative sentences affected because the associated frowning expression could not be made.

The studies discussed so far on facial expression and mimicry illustrate how sensorimotor simulation occurs and under what conditions this may or may not happen. This effect is specific to the emotion that can be displayed on the face and the emotion that is depicted in or activated with the stimuli materials. The effects discussed so far were online effects where the observation and display of emotions occurred while processing emotional expressions on a face, or valenced information in sentences. A few studies have also examined offline effects of facial expression, supporting the claim that simulation may work offline when the bodily state no longer is relevant for a cognitive task to be performed.

Specifically, studies have assessed the effects of manipulations by having participants remember or retrieve emotional information after the task was completed (Schnall and Laird, 2003; Halberstadt et al., 2009). A study by Halberstadt et al. (2009) included pictures of ambiguous facial expressions that were paired with an emotion concept, such as “happy,” or with a concept unrelated to emotion, such as “reliable.” Afterward, faces encoded with the happy concepts were remembered as happier than in the learning phase whereas no such effect was demonstrated for the concepts that were unrelated to emotion (Halberstadt et al., 2009). The encoding process thus was relevant for later memory when the pictures of the faces and the labels of the concepts were no longer available for comparison. In another study, participants practiced facial expressions and body postures based on emotional cues (Schnall and Laird, 2003). After this practice phase, participants responsive to personal cues recalled more life events that were emotionally congruent (positive memories to an upright body posture and smiling facial expression) than incongruent with the practiced expressions.

Both studies demonstrated offline simulation because once an emotion-congruent association of facial or bodily expression with emotion concepts was established, memory for encoded or newly retrieved materials was higher under congruent than under incongruent conditions. These studies on facial mimicry also underscore the causal role in emotion processing. An emotional stimulus is needed in order for the simulation to occur or be blocked. Otherwise, simulation of the emotional expression on the face would be irrelevant. Moreover, if the emotion cannot be simulated on the face because of a physical (pen) or mental (suppression) prevention of emotion to be shown, the benefits as a result of the compatibility effects disappear. Finally, the simulation mechanism may work offline, after a bodily state has occurred. This was shown in the studies demonstrating benefits of facial mimicry in memory tasks in which earlier encoded information under simulated conditions had to be retrieved at a later point in time when the manipulation of the body was no longer relevant.

Facial mimicry was the last domain that demonstrated sensorimotor simulation, in particular its effect on emotion processing and within a social context. Together, these six domains, language comprehension, gestures, autobiographical memory, expertise, problem solving, and facial mimicry, support an account of sensorimotor simulation to explain embodiment effects in specific ways. Now, it is time to take stock of what this empirical support in those domains has brought us. How stable and general is sensorimotor simulation?

## Conclusion

Some researchers claim that the degree of simulation required for a task is inversely related to the abstraction needed for it (Myachykov et al., 2014). In this view, the more abstract the process, the more offline it is, and the more stable it is. In contrast, online processes are less stable because of their sensitivity to individual differences in the sensorimotor experience. Although this may be a promising notion that could be developed further, our review shows rather how universal sensorimotor simulation is, as much for abstract and offline processes as for concrete and online processes, by accounting for a wide variety of embodiment effects in various domains. It remains to be seen whether online processes are less stable than offline processes and how sensitive online processes are to individual differences in the sensorimotor experience. Based on the discussion of the studies on online processes in this review, there is no evidence that online processes are less stable than offline processes.

Interference of embodiment was demonstrated in research on language comprehension, gestures, and problem solving. This phenomenon occurs when the same neural circuits are engaged for the sensorimotor as the cognitive processes in an experiment. At this point, it is not entirely clear exactly how the interference occurs and whether it is strictly a capacity problem or a dual engagement problem. It appears that involvement in one task leaves insufficient resources for the other and that this interferes with simulation. This was also found in some of the gesture studies that demonstrated negative effects of gestures among children with poor general language skills (Post et al., 2013). For this group, gestures increased cognitive load and had a negative effect on performance. Further examination of these conditions is essential to a deeper understanding of how sensorimotor simulation operates.

As said before, this overview of research on sensorimotor simulation in various domains is by no means exhaustive. Examples from different domains were chosen to illustrate this mechanism and specific claims operating under this mechanism. This review can be considered a first step toward an approach that further defines conditions under which embodiment occurs or not, and how they depend on previous experiences and skills, affect emotion processing, and operate in social situations. The evidence so far points in the direction of congruence/compatibility being a necessary condition for embodiment to occur and for engagement of similar systems to hinder embodiment. Only in this context can offline simulation and emotion processing be accomplished. The role of simulation in social interactions requires further study. Only support from one domain was discussed in this review.

Future research should also start to address questions regarding individuals who may be less able or susceptible to the effects of the body. How does sensorimotor simulation work for individuals with depression, aphasia or dementia? Would children be more or less affected by manipulations of the body, or does it not make a difference? For example, children with poor language skills may not benefit as much from body manipulations, such as gestures, compared to children with average to good language skills (Post et al., 2013). Likewise, older adults may not benefit as much from embodied encoding compared to young adults when their memories that are being retrieved, are more remote (Dijkstra et al., 2007). On the other hand, in the posture study (Dijkstra et al., 2007), older adults demonstrated as much facilitation from congruent postures as younger adults.

With these studies, domains, and claims, we have now a better understanding of one of the major mechanisms underlying embodiment; sensorimotor simulation. The future holds promise for further exploration and specification of the claims associated with this mechanism and gathering empirical evidence to support these claims.

### Conflict of Interest Statement

The authors declare that the research was conducted in the absence of any commercial or financial relationships that could be construed as a potential conflict of interest.
